# Food insecurity and sociodemographic determinants: a cross-sectional analysis from Türkiye nutrition and health survey (TNHS) – 2017

**DOI:** 10.1186/s12889-026-27388-z

**Published:** 2026-04-20

**Authors:** Simay Kundakçı, Güleren Sabuncular, Elif Akbaş, Sümeyye Gezer, Şule Aktaç

**Affiliations:** Department of Nutrition and Dietetics, Faculty of Health Sciences, Marmara University, Istanbul, Türkiye

**Keywords:** Food insecurity, Sociodemographic factors, Türkiye, Survey

## Abstract

**Background:**

Food insecurity is a major public health concern with multidimensional determinants and varying severity levels. Understanding its sociodemographic and regional drivers is essential for developing targeted interventions. This study aimed to examine the prevalence of mild, moderate, and severe food insecurity in Türkiye and to identify associated sociodemographic determinants using nationally representative data.

**Methods:**

This cross-sectional study analyzed secondary data from the Türkiye Nutrition and Health Survey (TNHS-2017), which included 12,986 individuals aged 15 years or older. Food insecurity was assessed using the Food Insecurity Experience Scale (FIES) and categorized as mild, moderate, or severe. Associations between the severity of food insecurity and sociodemographic factors were examined using multinomial logistic regression, with food-secure individuals as the reference group.

**Results:**

Perceived household financial situation emerged as the strongest determinant across all severity levels, followed by low educational attainment (*p* < 0.001). Compared with men, women had a higher risk of mild and moderate food insecurity (*p* < 0.001) but a lower risk of severe food insecurity (*p* < 0.001). Older age showed a protective effect, with significantly reduced risks among individuals aged 50–64 years and those aged 65 years and older (*p* < 0.001). Relative to higher education levels, all lower education levels were associated with an increased risk of food insecurity, reaching a 5.7-fold increase for severe food insecurity (*p* < 0.001). While marital status showed no significant association for mild and moderate food insecurity, never-married and widowed/divorced/separated individuals had a higher risk of severe food insecurity (*p* < 0.05). Significant regional disparities were observed, with the highest risks concentrated in Northeastern Anatolia, while some regions exhibited protective effects.

**Conclusion:**

Food insecurity in Türkiye is shaped by intersecting socioeconomic and regional factors that vary in severity. Income inadequacy and low education represent the most critical risk factors, while advanced age appears to be protective. These findings underscore the need for region-specific and equity-oriented policies that address the structural determinants of food insecurity.

**Supplementary Information:**

The online version contains supplementary material available at 10.1186/s12889-026-27388-z.

## Background

In the 1948 Universal Declaration of Human Rights, the right to access food, alongside political and economic rights, was emphasized as one of the most fundamental human rights. The World Health Organization (WHO) defines food security as the right of individuals to develop healthy eating habits by accessing reliable, sustainable, affordable, and high-quality food [1] Similarly, the Food and Agriculture Organization (FAO) states that food security is achieved when all people, at all times, have physical and economic access to sufficient, safe, and nutritious food that meets their dietary needs and food preferences for an active and healthy life [2].

Food insecurity is defined as uncertain or inadequate access to sufficient and safe nutrition, or limited ability to obtain such nutrition through socially appropriate means [3].

This multidimensional construct is traditionally anchored on four pillars: food availability, accessibility, utilization, and stability [4].

While global frameworks provide a standard definition, food insecurity is increasingly recognized as a complex social determinant of health, heavily influenced by localized socioeconomic, demographic, and environmental dynamics [5, 6]. In the context of Türkiye, the urgency of addressing food insecurity has intensified due to shifting economic landscapes and demographic pressures. According to the 2022 Global Food Security Index (GFSI), Türkiye ranks 49th among 113 countries. Although there were modest improvements in quality, safety, and sustainability dimensions between 2012 and 2022, sharp increases in food prices have significantly eroded the “affordability” dimension, offsetting overall progress [7]. Despite its growing importance, empirical evidence regarding the specific sociodemographic determinants of food insecurity in Türkiye remains fragmented. Recent studies have begun to explore these dynamics, focusing on specific populations such as the elderly [8, 9]. Syrian refugees [10] or general household dynamics using longitudinal data [11]. However, there is a notable research gap in comprehensive, nationally representative analyses that utilize standardized tools like the Food Insecurity Experience Scale (FIES) to evaluate the severity of food insecurity across diverse Turkish regions and sociodemographic strata. Understanding these domestic patterns is critical for aligning national public health policies with the Sustainable Development Goals, specifically the target to end hunger and ensure food access for vulnerable groups by 2030 [12]. Food insecurity is not merely a lack of calories; it is frequently associated with micronutrient deficiencies, poor dietary quality, and adverse health outcomes across the lifespan [13]. In adults, it is linked to an increased risk of obesity, depression, and chronic conditions such as diabetes and cardiovascular diseases, while in children, it can manifest as stunting and academic difficulties [14]. These risks are disproportionately borne by disadvantaged groups, including women, families with children, and those with limited financial resources [5, 11].

The Türkiye Nutrition and Health Survey (TNHS) is a national study representative of the Turkish population, designed to monitor nutritional habits, health status, and related biochemical indicators. Conducted by the Ministry of Health in 2010 and 2017, it serves as the most comprehensive data source for evaluating the nation’s nutritional landscape [15]. By utilizing the TNHS-2017 dataset, this study addresses the aforementioned gap in the literature by providing a detailed analysis of food insecurity levels. The study aims to examine the prevalence of food insecurity in Türkiye and identify its associations with key sociodemographic and regional determinants. By identifying these predictors, the findings intend to provide a fundamental resource for developing evidence-based public health interventions and social policies tailored to the Turkish context.

## Materials and methods

### Research design and data source

A secondary data analysis was conducted in this study between September 2025 and November 2025, utilizing the TNHS-2017 data. The research hypothesis was formulated as follows: demographic characteristics (age, sex, marital status, education level, and income status) and geographical region of residence are associated with the level of food insecurity.

### Population and data collection

The population for the research consists of individuals aged ≥ 15 years in Türkiye. The selected sample from the TNHS-2017 was utilized, and 12,986 participants (both female and male) aged 15 years and older were included. In this study, data regarding the participants’ demographic characteristics and the FIES data from the TNHS-2017 dataset were used.

### Food insecurity experience scale (FIES)

The Food Insecurity Experience Scale (FIES) is an experience-based metric developed by the Food and Agriculture Organization (FAO) to assess individuals’ access to adequate food. It captures both quantitative and qualitative dimensions of food insecurity, including uncertainty about food access and reductions in food intake due to financial or resource constraints. The classification thresholds are defined according to FAO guidelines, which group items by increasing severity, from concerns about food access (mild), to compromised food quality and quantity (moderate), and finally to experiences of hunger and food deprivation (severe). Classification based on participants’ “Yes” responses indicates that a “Yes” answer to questions 1 through 3 signifies mild food insecurity, a “Yes” answer to questions 4 through 6 signifies moderate food insecurity, and a “Yes” answer to questions 7 and 8 signifies severe food insecurity [16–18]. In this study, food insecurity was categorized into four levels (food secure, mild, moderate, and severe) based on the number and pattern of affirmative responses, in accordance with FAO guidelines and previous literature.

### Statistical analysis

Descriptive statistics for the data were presented using frequency and percentage values for categorical variables. The Chi-square test was applied to examine the relationship between categorical variables (sex, age group, education level, marital status, income status, and NUTS-1 region) and the levels of food insecurity.

No missing data were observed in the variables included in the analysis; therefore, all cases were retained.

Although the dataset was derived from a nationally representative survey with a complex sampling design, sampling weights were not applied in the present analysis. This is because the primary objective of the study was to examine associations between variables rather than to produce nationally representative prevalence estimates.

The FIES has a multi-categorical structure consisting of food insecurity severity levels (food secure, mild, moderate, severe). Although the levels reflect increasing severity, it was anticipated that the effects of the covariates (sex, age, education, marital status, income, and NUTS-1 region) might differ in both direction and magnitude across severity levels. Therefore, multinomial logistic regression was preferred to estimate the simultaneous and category-specific effects of all covariates, allowing for the calculation of adjusted odds ratios (OR) and 95% confidence intervals (CI) separately for mild, moderate, and severe food insecurity relative to the reference category.

Although food insecurity levels are ordinal in nature, the proportional odds assumption required for ordinal logistic regression was formally tested using the Brant test. The results indicated that this assumption was violated (Omnibus test, *p* < 0.001). Therefore, multinomial logistic regression was considered more appropriate, as it does not rely on the proportional odds assumption and allows for greater flexibility in estimating category-specific associations.

Multinomial logistic regression analysis was conducted to identify the independent variables associated with food insecurity. In the analyses, the “food secure” group was used as the reference category. Reference categories for the independent variables were selected based on both the sample distribution and previous literature to facilitate meaningful comparisons across groups: male for sex, 19–49 years for age, higher education for education, married for marital status, able to make ends meet comfortably for income, and Istanbul for regional comparisons.

All analyses were conducted using Jamovi version 2.3.28 (The Jamovi Project, Sydney, Australia), and statistical significance was set at *p* < 0.05.

## Results

The study examined the relationships between individuals’ food insecurity status and their sociodemographic characteristics, and statistically significant differences were found across all variables based on chi-square tests (*p* < 0.001). A total of 12,986 individuals participated in the study, with 55.1% of them being female. Overall, 66.4% of participants were food secure, while 33.6% experienced some level of food insecurity (mild, moderate, or severe). The prevalence of severe food insecurity was 8.2% among women and 9.3% among men (*p* < 0.001). Across age groups, severe food insecurity was most frequently observed in individuals aged 19–49 years (9.2%) (*p* < 0.001). Food insecurity decreases as the level of education increases; the rate of severe food insecurity was 14.1% among illiterate individuals, compared to 3.2% among those with higher education (*p* < 0.001). Regarding marital status, food insecurity at any level was more prevalent among married individuals (34.8%) compared to never-married individuals (25.4%), while severe food insecurity was highest among widowed, divorced, or separated individuals (10.2%) (*p* < 0.001). Perceived household financial situation showed the strongest gradient with food insecurity. The prevalence of food insecurity increased markedly from 6.6% among individuals living comfortably to 70.8% among those unable to meet monthly expenses (*p* < 0.001). The highest food security was observed in the East Marmara (72.2%) and Aegean (70.4%) regions. In comparison, the highest rates of severe food insecurity were observed in the Southeastern Anatolia (13.2%) and Northeastern Anatolia (13.5%) regions (*p* < 0.001) (Table 1).

**Table 1:** Comparison of sociodemographic characteristics of individuals according to their food insecurity status (*n* = 12,986)

	Food Secure (*n* = 8618)	Mild (*n* = 1354)	Moderate (*n* = 1885)	Severe (*n* = 1129)	*p*
Sex					
Female	4565 (63.7)	823 (11.5)	1184 (16.5)	589 (8.2)	< 0.001
Male	4053 (69.6)	531 (9.1)	701 (12.0)	540 (9.3)	
Age (years)					
15–18	379 (72.1)	34 (6.5)	71 (13.5)	42 (8.0)	< 0.001
19–49	4820 (67.0)	713 (9.9)	998 (13.9)	662 (9.2)	
50–64	1888 (62.8)	352 (11.7)	506 (16.8)	261 (8.7)	
≥ 65	1531 (67.7)	255 (11.3)	310 (13.7)	164 (7.3)	
Educational status					
Illiterate	590 (48.0)	168 (13.7)	299 (24.3)	173 (14.1)	< 0.001
Literate	276 (52.0)	71 (13.4)	111 (20.9)	73 (13.7)	
Primary education	2707 (59.6)	581 (12.8)	796 (17.5)	459 (10.1)	
Secondary education	1196 (64.9)	203 (11.0)	266 (14.4)	178 (9.7)	
High school	1931 (74.8)	205 (7.9)	273 (10.6)	173 (6.7)	
Higher education	1917 (85.0)	126 (5.6)	140 (6.2)	73 (3.2)	
Marital status					
Never married	1617 (74.6)	157 (7.2)	217 (10.0)	176 (8.1)	< 0.001
Married	5975 (65.3)	1006 (11.0)	1390 (15.2)	783 (8.6)	
Widowed/divorced/living separately	1025 (61.6)	191 (11.5)	278 (16.7)	170 (10.2)	
Perceived household financial situation					
Lives comfortably	2592 (93.4)	71 (2.6)	73 (2.6)	39 (1.4)	< 0.001
Lives without serious financial hardship	2810 (83.0)	233 (6.9)	238 (7.0)	106 (3.1)	
Barely meets monthly expenses	2545 (54.7)	716 (15.4)	945 (20.3)	447 (9.6)	
Unable to meet monthly expenses	604 (29.2)	324 (15.7)	610 (29.5)	528 (25.6)	
Does not know	66 (63.5)	10 (9.6)	19 (18.3)	9 (8.7)	
NUTS − 1 regions					
İstanbul	1297 (70.1)	166 (9.0)	234 (12.7)	152 (8.2)	< 0.001
West Marmara	535 (68.9)	57 (7.3)	110 (14.2)	74 (9.5)	
Aegean	1569 (70.4)	175 (7.9)	305 (13.7)	180 (8.1)	
East Marmara	966 (72.2)	156 (11.7)	137 (10.2)	79 (5.9)	
West Anatolia	913 (69.8)	170 (13.0)	156 (11.9)	69 (5.3)	
Mediterranean	1102 (62.2)	227 (12.8)	279 (15.8)	163 (9.2)	
Central Anatolia	459 (67.5)	57 (8.4)	109 (16.0)	55 (8.1)	
West Black Sea	572 (67.1)	89 (10.4)	111 (13.0)	81 (9.5)	
East Black Sea	295 (69.4)	44 (10.4)	41 (9.6)	45 (10.6)	
Northeast Anatolia	136 (49.6)	33 (12.0)	68 (24.8)	37 (13.5)	
Middle East Anatolia	306 (55.3)	62 (11.2)	114 (20.6)	71 (12.8)	
Southeast Anatolia	468 (50.3)	118 (12.7)	221 (23.8)	123 (13.2)	

Chi-square test

**Fig. 1:**
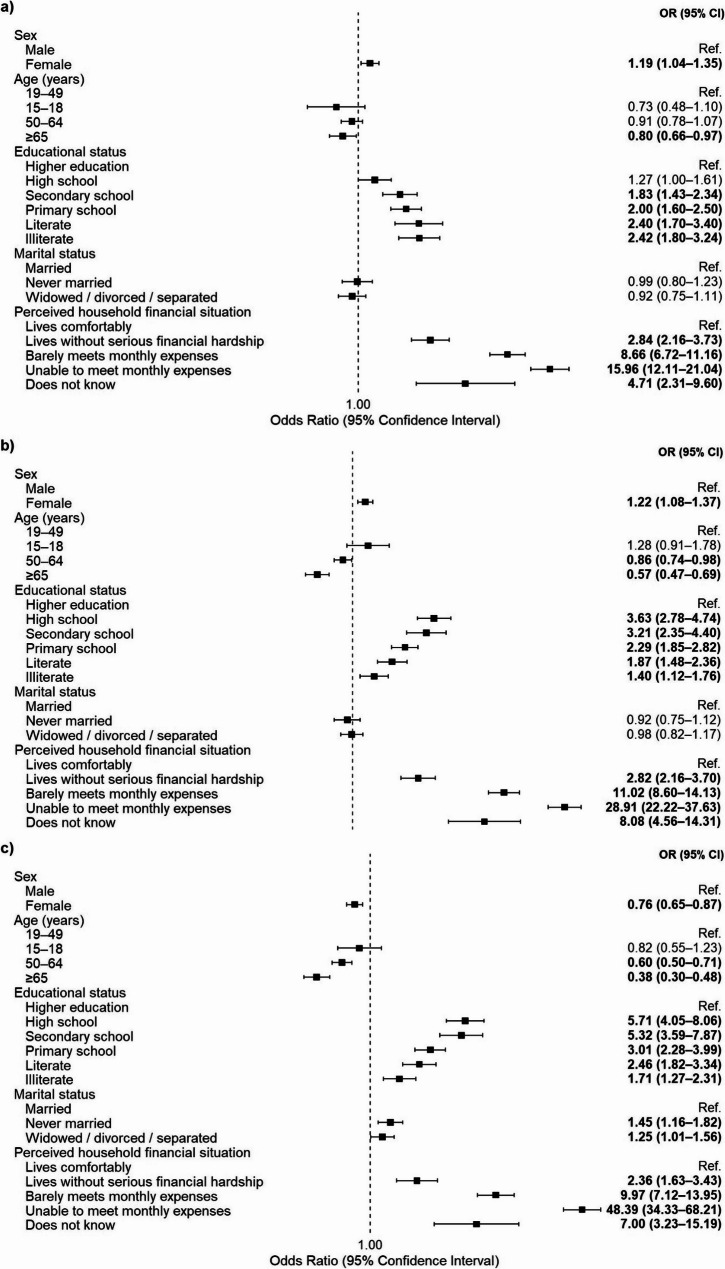
**a** Mild Food Insecurity, (**b**) Moderate Food Insecurity, (**c**) Severe Food Insecurity

Sociodemographic factors associated with mild, moderate, and severe food insecurity are presented in Fig. 1, with detailed results provided in Supplementary Material 1.

Perceived household financial situation emerged as the strongest determinant across all levels of food insecurity. The risk of mild and moderate food insecurity was approximately 1.2 times higher in women compared to men, but 0.9 times lower for severe food insecurity (*p* = 0.009 and *p* < 0.001, respectively). The risk of mild food insecurity decreases by approximately 0.8 times in the 65 + age group (*p* < 0.05). For moderate food insecurity, the risk decreased by 0.86 times in the 50–64 age group (*p* < 0.05) and by 0.57 times in the 65 + age group (*p* < 0.001). Similarly, the risk of severe food insecurity decreased by 0.60 times in the 50–64 age group (*p* < 0.001) and by 0.38 times in individuals aged 65 years and older (*p* < 0.001). Compared to higher education graduates, the risk of food insecurity increased across all categories for other educational levels, with this risk ranging from 1.4 to 5.7 times higher (*p* < 0.001). Furthermore, while the highest risk for mild food insecurity was observed in both illiterate and literate individuals (2.4 times), the risk of moderate (3.6 times) and severe (5.7 times) food insecurity was higher among high school graduates (*p* < 0.001). Regarding marital status, although no significant difference was found in the mild and moderate categories, the risk of severe food insecurity was determined to increase by approximately 1.5 times in never-married individuals, and by 1.25 times in those who were widowed, divorced, or separated (*p* < 0.05). Perceived household financial situation emerged as the strongest determinant in all models; compared to those who can make ends meet comfortably, the risk increased by 2.8–16 times for the mild level (*p* < 0.001), 2.8–29 times for the moderate level (*p* < 0.001), and 2.4–48 times for the severe level (*p* < 0.001).

**Fig. 2:**
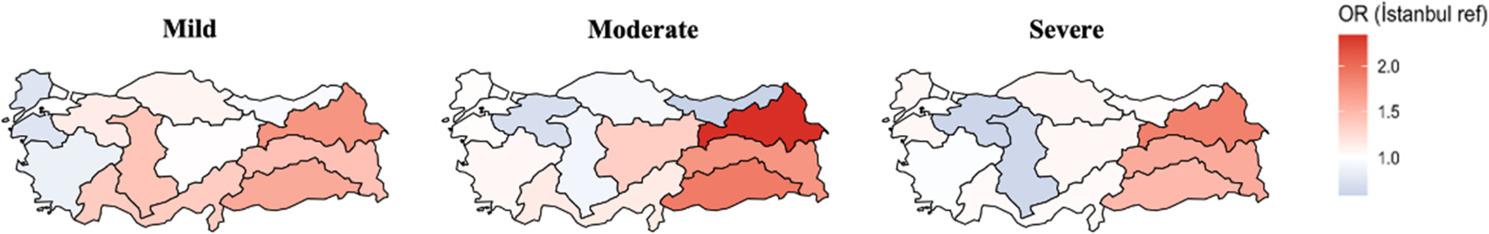
Food insecurity levels by NUTS-1 in Türkiye

When Istanbul was taken as the reference, it was determined that the risk of food insecurity differed significantly among Türkiye’s NUTS-1 regions. For mild food insecurity, the highest risk was observed in the Northeastern Anatolia Region (1.74 times), and the lowest risk was in the Mediterranean Region (1.36 times). The risk of moderate food insecurity was determined to be highest in Northeastern Anatolia (2.34 times) and lowest in Eastern Black Sea (0.58 times). The risk of severe food insecurity was found to increase by 1.87 times in the Northeastern Anatolia Region, whereas it decreased by 0.60 times in the Eastern Marmara Region (*p* < 0.05) (Fig. 2).

## Discussion

This study reveals that one-third of individuals aged 15 years and older in Türkiye experience food insecurity at varying levels. It was determined that the sociodemographic characteristics with the greatest impact on food insecurity are education status, perceived household financial situation, and the region of residence. Higher education and perceived household financial situation, as well as residing in Western regions, were found to reduce the risk of food insecurity. A study using data from 11,863 households in our country found that approximately 35% of the sample experienced food insecurity, and the probability of food insecurity decreased significantly as the level of education, health status, and income increased [15]. Our findings align with recent longitudinal analyses in Türkiye, which emphasize that household head’s education and income stability are the most resilient buffers against shifting food prices [11].

It was determined that the risk of mild and moderate food insecurity was significantly higher in women, while men exhibited a higher risk for severe food insecurity. The literature suggests that women’s greater exposure to food insecurity compared to men is closely linked to limited opportunities for education, income, and assets; exclusion from employment and decision-making processes; the burden of care; and prevailing gender norms [19–22]. While education is often reported as a protective factor against food insecurity [19, 23], our findings suggest that the association between gender and food insecurity persists independently of educational differences. Conversely, increasing household size and the number of children heighten the risk of food insecurity for women [19–21]. Furthermore, gender-based inequalities in income and employment sectors [20, 22] and a lack of representation further deepen this vulnerability [23–25]. According to the Turkish Statistical Institute (TÜİK)’s Women in Statistics – 2017 report, the employment rate for women is less than half that of men, and the labor force participation rate increases with the level of education [26]. The higher vulnerability of women in mild and moderate food insecurity can be explained by their lower employment rates and their responsibility for food procurement and meal preparation [27]. Conversely, the finding that men face a higher risk of severe food insecurity may be related to their being primary income providers for the household and thus being more severely affected by unemployment and irregular incomes [28].

The risk of both moderate and severe food insecurity was observed to decrease significantly in the 50–64 age group and the ≥ 65 age group. Similarly, the literature reports that the risk of food insecurity for older individuals is reduced through various social and economic mechanisms [29–32]. Retirement pensions, social assistance, and food support programs specifically designed for older people have been shown to mitigate food insecurity by increasing household income or compensating for income losses [29–32]. However, recent multicenter studies in Türkiye caution that while older adults may have stable pensions, food insecurity in this group is strongly associated with geriatric syndromes like frailty and malnutrition [8, 9].

In addition, the support older individuals receive from their children or family members plays a protective economic role [33–35]. Intra-family support networks contribute to the food supply by improving the welfare of older individuals and reducing their risk of food insecurity, especially in rural areas [36]. Furthermore, the fact that a portion of the older population sustains itself through their own production in rural areas can be considered a supporting factor for this finding [37].

The relationship between education level and food insecurity exhibited a complex pattern, where the protective effect was most pronounced at the highest level of attainment. While the risk of food insecurity generally increased as educational levels decreased, this association did not follow a strictly linear or staggered trajectory across all severity levels, as might be expected from traditional human capital theories. For mild food insecurity, the increase in risk was relatively limited, becoming more noticeable only in groups with secondary education or lower. However, a substantial widening of the educational gap was observed for moderate and severe food insecurity. Specifically, for moderate food insecurity, the risk increased more than threefold among high school and secondary education graduates compared to those with higher education. This disparity was most pronounced for severe food insecurity, where individuals with high school or secondary education faced up to five times the risk of their higher-educated counterparts.

These findings may suggest the presence of a threshold-like effect, whereby the protective benefits of education become more evident primarily at higher levels of attainment (i.e., university level). Although the literature generally indicates that increasing educational attainment is associated with lower food insecurity risk through improved nutrition literacy, resource management, and income-generating capacity [19, 21, 23], our results point to a more nuanced relationship. Education is expected to contribute to food security through enhanced economic productivity and access to stable employment opportunities [38]. However, the elevated risk observed even among high school graduates suggests that intermediate levels of education may not provide sufficient economic stability to buffer against moderate or severe food insecurity in the current economic context. 

This interpretation is supported by national data from the TÜİK Income and Living Conditions Survey (2017), which shows a marked reduction in poverty rates only at the higher education level (1.5%), compared to 5.5% among high school graduates and 25.4% among illiterate individuals [39]. Increasing the level of education plays a critical role not only in individual well-being but also in ensuring food security at the household level. he absence of a clear gradient between intermediate education levels in our findings may reflect labor market dynamics in Türkiye, where the economic returns of secondary and high school education are relatively limited. In addition, education may influence food security through behavioral pathways, including food access strategies, dietary choices, and health-related knowledge [20]. Therefore, while increasing educational attainment remains important, these findings indicate that the relationship between education and food insecurity is not uniform across levels. From a public health perspective, this suggests the need for targeted interventions addressing the vulnerabilities of individuals with intermediate education levels, alongside broader efforts to improve access to higher education and economic opportunities.

No significant association was observed between marital status and mild or moderate food insecurity; however, a significant association was identified for severe food insecurity. Marriage facilitates food access and contributes to the efficient use of resources through mechanisms such as income sharing, joint management of expenditures, and the strengthening of social support networks [40, 41]. Studies conducted in Türkiye have reported that marriage is a determinant of spouses’ employment status, household income stability, and food access [15, 42]. Furthermore, the social resilience provided by marital relationships can mitigate the negative effects of economic hardship by offering both emotional and material support [41]. Conversely, single-person households cannot share income and are forced to bear fixed expenses alone, making them more vulnerable to income fluctuations [43].

Among individuals who are widowed or divorced, the reduction in household income, the weakening of social support, and the psychosocial burden of living alone can deepen food insecurity. Existing studies demonstrate that widowed, divorced, or single individuals are more frequently represented in severe food insecurity categories [44, 45]. The increasing divorce rates and decreasing marriage rates in Türkiye [46] may be associated with changes in household structures, including a potential increase in single-person households. Household income status was identified as the strongest determinant across all levels of food insecurity, with the risk of food insecurity increasing sharply as perceived financial hardship intensified. While mild food insecurity can often be managed through minor dietary adjustments, moderate and severe food insecurity represent deeper structural problems reflecting fundamental income inadequacy. In a study conducted in Iran, the risk of food insecurity among households in the lowest income group was reported to be 6.4 times higher than that of households in the highest income group [47].

This strong relationship between income and food insecurity can be explained by multiple mechanisms that collectively shape the household’s capacity to access food. Low income directly limits the capacity of households to procure sufficient quantity and quality of food, thereby reducing purchasing power. According to household budget analyses conducted in Türkiye, when purchasing power decreases, a larger portion of income is allocated to food, but the quality and quantity of food decline [48].

While high-income households can overcome temporary food access problems through savings, credit, or social support networks, low-income households lack these buffer mechanisms. Consequently, low-income individuals are forced to resort to short-term strategies to cope with food insecurity, such as consuming lower-quality foods, reducing the number of meals, or seeking emergency food assistance [49]. A study conducted in Nigeria supports the association between household income and food security, indicating that higher income levels are linked to improved food security outcomes [50].

According to TÜİK household consumption expenditure data for 2017, households in the lowest income quintile allocated 28.6% of their budget to food and non-alcoholic beverages, while this rate dropped to 14.6% in the highest income quintile [39]. As income decreases, the burden of food expenditure on the budget rises, and low-income households become more vulnerable to price fluctuations. Moreover, when assessing poverty dynamics, it is emphasized that formal income metrics alone do not fully reflect the risk of food insecurity. This is because non-food essential expenditures and increasing debt burdens reduce the disposable income available for food, thereby diminishing real purchasing power [51].

Beyond individual-level factors, food insecurity is also influenced by broader economic dynamics such as food price fluctuations and income instability. These interacting processes may further exacerbate existing sociodemographic inequalities in food access, suggesting that the patterns observed in this study should be interpreted within a wider structural context [52].

The analysis of the study by NUTS-1 regions reveals that the risk of food insecurity is significantly higher in the Northeastern, Eastern, and Southeastern Anatolia regions compared to Istanbul. At the same time, it is relatively lower in the Aegean and Marmara regions. Istanbul, Aegean, and Marmara regions enhance market access and economic resilience by serving as centers for industrial and service production. In contrast, in the Eastern areas, low-income levels, limited production diversity, and migration tendencies reinforce the risk of food insecurity [53–57].

The reliance on agriculture for livelihood in the East and Southeast increases vulnerability to production conditions and market fluctuations, thereby creating a structural fragility in terms of food access and nutrition [55, 58]. At the core of these regional differences lies the reciprocal interaction of socioeconomic, labor market, and spatial factors. Limitations in access to education and health services, particularly in the Eastern regions, weaken individuals’ capacity for food access and adaptation [59, 60]. Urbanization and migration processes, however, demonstrate a bidirectional effect. While improving the status of some households through income transfers and job connections, they also deepen regional inequalities by limiting the rural population’s access to services [54, 60].

From a public health and policy perspective, these findings provide important insights for strengthening existing food security interventions in Türkiye. Although multiple mechanisms are already in place—such as agricultural subsidies, social assistance programs, and food waste reduction initiatives—these policies are often implemented at a broad national level.

The findings of this study indicate that food insecurity is closely associated with sociodemographic characteristics including education level, perceived household financial situation, gender, and regional disparities. Therefore, policy responses may be more effective if they incorporate a targeted, sociodemographic approach, rather than relying solely on universal strategies.

In particular, individuals with lower and intermediate levels of education, those experiencing financial hardship, women, and populations living in Eastern and Southeastern regions appear to be at greater risk. Tailoring interventions to these high-risk groups—such as region-specific social assistance, gender-sensitive support programs, and policies aimed at improving economic stability—may enhance the effectiveness of existing measures.

These findings highlight the importance of integrating sociodemographic risk profiles into policy design, ensuring that food security interventions are not only available but also appropriately targeted to the most vulnerable populations. Future research using longitudinal data would further support the development of more responsive and evidence-based policies in Türkiye.

### Limitations and strengths

The study has several limitations. The cross-sectional design of the research data does not permit the interpretation of relationships between variables at a causal level. As the study relies on secondary data analysis, the researchers had no control over the data collection process, and the variables that could be analyzed were restricted to the scope of the TNHS-2017 dataset. Since the FIES scale is based on self-report, it may lead to measurement errors, such as recall bias, which could potentially cause food insecurity levels to be reported as either lower or higher than the actual levels. Furthermore, some additional determinants, such as household structure, nutritional intake, or health status, could not be included in the model due to the constraints of the dataset.

Despite these methodological limitations, the study’s reliance on the large, representative sample of the TNHS-2017 data increases the generalizability of the results. The assessment of food insecurity using the FIES scale developed by the FAO strengthens the international comparability of the measurements. The multidimensional examination of sociodemographic variables and the estimation of separate risk predictions for each severity level of food insecurity using multinomial logistic regression enhances the analytical depth of the study. Moreover, the findings—which reveal regional and socioeconomic disparities—serve as an important guide for public health policies.

Future research should consider longitudinal study designs to better capture temporal changes and clarify potential causal relationships between sociodemographic factors and food insecurity. In addition, incorporating a broader range of variables—such as household composition, dietary intake, and health status—would provide a more comprehensive understanding of the determinants of food insecurity.

## Conclusion

Food insecurity in Türkiye is shaped by interconnected determinants that vary in severity. The findings reveal that insufficient income and low educational attainment constitute the strongest risk areas; advanced age is protective, while sex and marital status exhibit effects that shift direction based on severity. The concentration of risk in the Eastern and Southeastern Anatolia regions highlights the need for region-focused strategies. In this context, initiatives such as nutrition education and nutrition literacy programs, regulations aimed at balancing female employment, and projects that strengthen rural-urban linkages should be developed. Addressing remaining gaps will require longitudinal monitoring of transitions between severity levels, rigorous evaluation of program effectiveness, and causal analyses that examine how gender, education, income, and regional disparities influence the risk of food insecurity.

## Supplementary Information

Supplementary Material 1.

## Data Availability

The dataset used in this study was obtained from the Türkiye Nutrition and Health Survey (TNHS) 2017 conducted by the Republic of Türkiye Ministry of Health. As the dataset is not part of a public repository, it does not have an accession number. The datasets analyzed during the current study are not publicly available but can be accessed upon reasonable request and with permission from the relevant authorities.

## Abbreviations

FAO: Food and Agriculture Organization
FIES: Food Insecurity Experience Scale
GFSI: Global Food Security Index
SOFI: The State of Food Security and Nutrition in the World
TNHS: Türkiye Nutrition and Health Survey
TÜİK: Turkish Statistical Institute
WHO: World Health Organization
