# Supplementary material for: Food insecurity and sociodemographic determinants: a cross-sectional analysis from Türkiye nutrition and health survey (TNHS) – 2017
Source: BMC Public Health. 2026 Apr 20;26:1769. doi: 10.1186/s12889-026-27388-z (PMC13227847; doi:10.1186/s12889-026-27388-z)
Supplement: Supplementary file 1 — Supplementary Material 1. [file 12889_2026_27388_MOESM1_ESM.docx]

**Table 1.** Sociodemographic determinants associated with mild food insecurity

|  | Exp(B) | Lower Bound | Upper Bound | Sig. |
| --- | --- | --- | --- | --- |
| **Sex** |  |  |  |  |
| Female | . | . | . | . |
| Male | 1.189 | 1.045 | 1.354 | **0.009** |
| **Age (years)** |  |  |  |  |
| 19-49 | . | . | . | . |
| 15-18 | 0.729 | 0.481 | 1.104 | 0.136 |
| 50-64 | 0.913 | 0.782 | 1.067 | 0.253 |
| ≥65 | 0.801 | 0.659 | 0.974 | **0.026** |
| **Educational status** |  |  |  |  |
| Illiterate | . | . | . | . |
| Literate | 1.269 | 0.999 | 1.612 | .051 |
| Primary education | 1.828 | 1.427 | 2.342 | **<0.001** |
| Secondary education | 2.001 | 1.605 | 2.495 | **<0.001** |
| High school | 2.404 | 1.700 | 3.398 | **<0.001** |
| Higher education | 2.420 | 1.805 | 3.244 | **<0.001** |
| **Marital status** |  |  |  |  |
| Never married | . | . | . | . |
| Married | 0.988 | 0.796 | 1.227 | 0.915 |
| Widowed / divorced/living separately | 0.917 | 0.754 | 1.114 | 0.383 |
| **Perceived household financial situation** |  |  |  |  |
| Lives comfortably | . | . | . | . |
| Lives without serious financial hardship | 2.837 | 2.161 | 3.725 | **<0.001** |
| Barely meets monthly expenses | 8.659 | 6.721 | 11.156 | **<0.001** |
| Unable to meet monthly expenses | 15.962 | 12.108 | 21.043 | **<0.001** |
| Does not know | 4.710 | 2.310 | 9.599 | **<0.001** |
| **NUTS -1 regions** |  |  |  |  |
| İstanbul | . | . | . | . |
| West Marmara | 0.748 | 0.538 | 1.040 | 0.084 |
| Aegean | 0.850 | 0.673 | 1.074 | 0.173 |
| East Marmara | 1.136 | 0.890 | 1.451 | 0.306 |
| West Anatolia | 1.405 | 1.104 | 1.789 | **0.006** |
| Mediterranean | 1.357 | 1.083 | 1.701 | **0.008** |
| Central Anatolia | 1.011 | 0.724 | 1.413 | 0.946 |
| West Black Sea | 1.067 | 0.799 | 1.425 | 0.659 |
| East Black Sea | 0.946 | 0.652 | 1.373 | 0.770 |
| Northeast Anatolia | 1.739 | 1.122 | 2.697 | **0.013** |
| Middle East Anatolia | 1.433 | 1.026 | 2.003 | **0.035** |
| Southeast Anatolia | 1.599 | 1.215 | 2.106 | **<0.001** |

Multinomial logistic regression

**Table 2.** Sociodemographic determinants associated with moderate food insecurity

|  | **Exp(B)** | **Lower Bound** | **Upper Bound** | **Sig.** |
| --- | --- | --- | --- | --- |
| **Sex** |  |  |  |  |
| Female | . | . | . | . |
| Male | 1.220 | 1.084 | 1.373 | **<0.001** |
| **Age (years)** |  |  |  |  |
| 19-49 | . | . | . | . |
| 15-18 | 1.276 | 0.913 | 1.782 | 0.153 |
| 50-64 | 0.856 | 0.744 | 0.985 | **0.029** |
| ≥65 | 0.570 | 0.474 | 0.686 | **<0.001** |
| **Educational status** |  |  |  |  |
| Illiterate | . | . | . | . |
| Literate | 3.631 | 2.781 | 4.740 | **<0.001** |
| Primary education | 3.215 | 2.348 | 4.403 | **<0.001** |
| Secondary education | 2.287 | 1.855 | 2.820 | **<0.001** |
| High school | 1.866 | 1.475 | 2.361 | **<0.001** |
| Higher education | 1.404 | 1.120 | 1.761 | **0.003** |
| **Marital status** |  |  |  |  |
| Never married | . | . | . | . |
| Married | 0.916 | 0.747 | 1.121 | 0.394 |
| Widowed / divorced/living separately | 0.982 | 0.824 | 1.171 | 0.838 |
| **Perceived household financial situation** |  |  |  |  |
| Lives comfortably | . | . | . | . |
| Lives without serious financial hardship | 2.824 | 2.157 | 3.698 | **<0.001** |
| Barely meets monthly expenses | 11.024 | 8.603 | 14.126 | **<0.001** |
| Unable to meet monthly expenses | 28.913 | 22.217 | 37.628 | **<0.001** |
| Does not know | 8.082 | 4.564 | 14.314 | **<0.001** |
| **NUTS -1 regions** |  |  |  |  |
| İstanbul | . | . | . | . |
| West Marmara | 1.016 | 0.777 | 1.330 | 0.905 |
| Aegean | 1.057 | 0.865 | 1.292 | 0.589 |
| East Marmara | 0.699 | 0.549 | 0.891 | **0.004** |
| West Anatolia | 0.904 | 0.714 | 1.146 | 0.405 |
| Mediterranean | 1.146 | 0.932 | 1.410 | 0.196 |
| Central Anatolia | 1.339 | 1.015 | 1.765 | **0.039** |
| West Black Sea | 0.929 | 0.712 | 1.213 | 0.588 |
| East Black Sea | 0.581 | 0.398 | 0.850 | **0.005** |
| Northeast Anatolia | 2.339 | 1.624 | 3.369 | **<0.001** |
| Middle East Anatolia | 1.744 | 1.312 | 2.318 | **<0.001** |
| Southeast Anatolia | 1.912 | 1.509 | 2.422 | **<0.001** |

Multinomial logistic regression

Table 3. Sociodemographic determinants associated with severe food insecurity

|  | Exp(B) | Lower Bound | Upper Bound | Sig. |
| --- | --- | --- | --- | --- |
| **Sex** |  |  |  |  |
| Female | . | . | . | . |
| Male | 0.755 | 0.653 | 0.873 | **<0.001** |
| **Age (years)** |  |  |  |  |
| 19-49 | . | . | . | . |
| 15-18 | 0.824 | 0.552 | 1.233 | 0.347 |
| 50-64 | 0.598 | 0.500 | 0.715 | **<0.001** |
| ≥65 | 0.376 | 0.297 | 0.477 | **<0.001** |
| **Educational status** |  |  |  |  |
| Illiterate | . | . | . | . |
| Literate | 5.712 | 4.049 | 8.059 | **<0.001** |
| Primary education | 5.315 | 3.588 | 7.873 | **<0.001** |
| Secondary education | 3.014 | 2.278 | 3.987 | **<0.001** |
| High school | 2.463 | 1.817 | 3.340 | **<0.001** |
| Higher education | 1.714 | 1.273 | 2.308 | **<0.001** |
| **Marital status** |  |  |  |  |
| Never married | . | . | . | . |
| Married | 1.454 | 1.159 | 1.824 | **0.001** |
| Widowed / divorced/living separately | 1.254 | 1.009 | 1.560 | **0.041** |
| **Perceived household financial situation** |  |  |  |  |
| Lives comfortably | . | . | . | . |
| Lives without serious financial hardship | 2.364 | 1.628 | 3.432 | **<0.001** |
| Barely meets monthly expenses | 9.970 | 7.124 | 13.952 | **<0.001** |
| Unable to meet monthly expenses | 48.391 | 34.331 | 68.208 | **<0.001** |
| Does not know | 7.005 | 3.231 | 15.187 | **<0.001** |
| **NUTS -1 regions** |  |  |  |  |
| İstanbul | . | . | . | . |
| West Marmara | 1.053 | 0.762 | 1.454 | 0.756 |
| Aegean | 0.969 | 0.756 | 1.242 | 0.804 |
| East Marmara | 0.604 | 0.446 | 0.820 | **0.001** |
| West Anatolia | 0.630 | 0.459 | 0.865 | **0.004** |
| Mediterranean | 1.032 | 0.799 | 1.331 | 0.811 |
| Central Anatolia | 1.052 | 0.737 | 1.503 | 0.779 |
| West Black Sea | 1.060 | 0.774 | 1.451 | 0.718 |
| East Black Sea | 1.022 | 0.691 | 1.512 | 0.915 |
| Northeast Anatolia | 1.871 | 1.196 | 2.924 | **0.006** |
| Middle East Anatolia | 1.586 | 1.126 | 2.233 | **0.008** |
| Southeast Anatolia | 1.484 | 1.111 | 1.983 | **0.008** |

Multinomial logistic regression
